# Supplementary material for: Mapping the evolution of fertility support policies in China: A content and instrumental analysis
Source: PLoS One. 2025 Oct 9;20(10):e0332137. doi: 10.1371/journal.pone.0332137 (PMC12510515; doi:10.1371/journal.pone.0332137)
Supplement: S1 Appendix — (ZIP) [file pone.0332137.s001.zip › S1 Appendix. 226 original policy documents/78-国务院关于印发“十三五”推进基本公共服务均等化规划的通知(FBM-CLI-2-290866).docx]

国务院关于印发“十三五”推进基本公共服务均等化规划的通知

发布部门： 国务院

发文字号：国发〔2017〕9号

发布日期：2017.01.23

实施日期：2017.01.23

时效性： 现行有效

效力级别： 国务院规范性文件

法规类别： 机关工作综合规定

国务院关于印发“十三五”推进基本公共服务均等化规划的通知

（国发〔2017〕9号）

各省、自治区、直辖市人民政府，国务院各部委、各直属机构：

　　现将《“十三五”推进基本公共服务均等化规划》印发给你们，请认真贯彻执行。

国务院

2017年1月23日

　　“十三五”推进基本公共服务均等化规划

　　基本公共服务是由政府主导、保障全体公民生存和发展基本需要、与经济社会发展水平相适应的公共服务。基本公共服务均等化是指全体公民都能公平可及地获得大致均等的基本公共服务，其核心是促进机会均等，重点是保障人民群众得到基本公共服务的机会，而不是简单的平均化。享有基本公共服务是公民的基本权利，保障人人享有基本公共服务是政府的重要职责。推进基本公共服务均等化，是全面建成小康社会的应有之义，对于促进社会公平正义、增进人民福祉、增强全体人民在共建共享发展中的获得感、实现中华民族伟大复兴的中国梦，都具有十分重要的意义。

　　本规划依据《中华人民共和国国民经济和社会发展第十三个五年规划纲要》编制，是“十三五”乃至更长一段时期推进基本公共服务体系建设的综合性、基础性、指导性文件。

　第一章　规划背景

　第一节　发展基础

　　“十二五”以来，我国已初步构建起覆盖全民的国家基本公共服务制度体系，各级各类基本公共服务设施不断改善，国家基本公共服务项目和标准得到全面落实，保障能力和群众满意度进一步提升。截至2015年，义务教育均衡发展深入推进，国民受教育机会显著增加，九年义务教育巩固率达到93%，进城务工人员随迁子女在流入地公办学校就读的比例超过80%；实施就业优先战略，公共就业创业服务和职业培训不断强化，全国就业人员达到77451万人，劳动者参加就业技能培训后就业率平均达70%以上；覆盖城乡的社会保障体系进一步健全，城乡居民养老保险制度实现整合，保障水平稳步提高，社会服务体系继续完善，临时救助制度全面实施，残疾人小康进程加快推进；基本公共卫生服务项目增加到12类，全民医保体系加快健全，基本医保参保率超过95%，大病保险覆盖全部城乡居民医保参保人员，国家基本公共卫生服务经费和城乡居民基本医疗保险补助标准分别提高到每人每年40元和380元，人民健康水平总体上达到中高收入国家平均水平；城镇保障性安居工程和农村危房改造力度加大，全国累计开工城镇保障性安居工程住房4013万套、其中改造棚户区住房2191万套，改造农村危房1794万户；现代公共文化服务体系建设积极推进，农村公共文化服务能力增强，全民健身活动蓬勃开展，广播、电视人口综合覆盖率均达到98%。

　　同时，我国基本公共服务还存在规模不足、质量不高、发展不平衡等短板，突出表现在：城乡区域间资源配置不均衡，硬件软件不协调，服务水平差异较大；基层设施不足和利用不够并存，人才短缺严重；一些服务项目存在覆盖盲区，尚未有效惠及全部流动人口和困难群体；体制机制创新滞后，社会力量参与不足。

　第二节　发展环境

　　“十三五”时期是全面建成小康社会的决胜阶段，我国发展仍处于可以大有作为的重要战略机遇期，完善国家基本公共服务体系、推动基本公共服务均等化水平稳步提升，面临新的机遇和挑战。

　　--经济进入新常态。经济增长从高速转向中高速，经济结构深度调整，发展动力加快转换，保民生兜底线的任务更加艰巨。同时民生持续改善也会为经济发展创造更多有效需求，为推进供给侧结构性改革提供强大内生动力。

　　--人口形成新结构。人口总量增长势头明显减弱，劳动年龄人口减少，人口老龄化加速，老年抚养比上升，新型城镇化推动城乡人口结构变化，对公共服务供给结构、资源布局、覆盖人群等带来较大影响。

　　--社会呈现新特征。社会结构深刻变动、利益格局深刻调整，人民群众的公平意识、民主意识、权利意识不断增强，合理引导社会预期、加快基本公共服务均等化任务更加艰巨。

　　--消费体现新需求。中等收入群体规模不断扩大，群众提高生活水平和改善生活质量的愿望更加强烈，消费需求更加多样化多层次，提高公共服务供给质量和水平的要求更加紧迫。

　　--科技孕育新突破。新一轮科技革命和产业变革正在兴起，移动互联网、物联网、大数据、云计算等技术快速发展，推动公共服务新业态不断发展、供给方式不断创新、服务模式更加丰富。

　第二章　指导思想和主要目标

　第一节　指导思想

　　高举中国特色社会主义伟大旗帜，全面贯彻党的十八大和十八届三中、四中、五中、六中全会精神，深入贯彻习近平总书记系列重要讲话精神和治国理政新理念新思想新战略，认真落实党中央、国务院决策部署，统筹推进“五位一体”总体布局和协调推进“四个全面”战略布局，牢固树立和贯彻落实新发展理念，坚持以人民为中心的发展思想，坚持以社会主义核心价值观为引领，从解决人民群众最关心最直接最现实的利益问题入手，以普惠性、保基本、均等化、可持续为方向，健全国家基本公共服务制度，完善服务项目和基本标准，强化公共资源投入保障，提高共建能力和共享水平，努力提升人民群众的获得感、公平感、安全感和幸福感，实现全体人民共同迈入全面小康社会。

　　--兜住底线，引导预期。立足基本国情，充分发挥基本公共服务兜底作用，牢牢把握服务项目，严格落实服务指导标准。坚持尽力而为、量力而行，合理引导社会预期，通过人人参与、人人尽力，实现人人共享。

　　--统筹资源，促进均等。统筹运用各领域各层级公共资源，推进科学布局、均衡配置和优化整合。加大基本公共服务投入力度，向贫困地区、薄弱环节、重点人群倾斜，推动城乡区域人群均等享有和协调发展。

　　--政府主责，共享发展。深化简政放权、放管结合、优化服务改革，划清政府与市场界限，增强政府基本公共服务职责，合理划分政府财政事权和支出责任，强化公共财政保障和监督问责。充分发挥市场机制作用，支持各类主体平等参与并提供服务，形成扩大供给合力。

　　--完善制度，改革创新。推进基本公共服务均等化、标准化、法制化，促进制度更加规范。加快转变政府职能，创新服务提供方式，消除体制机制障碍，全面提升基本公共服务质量、效益和群众满意度。

　第二节　主要目标

　　到2020年，基本公共服务体系更加完善，体制机制更加健全，在学有所教、劳有所得、病有所医、老有所养、住有所居等方面持续取得新进展，基本公共服务均等化总体实现。

　　--均等化水平稳步提高。城乡区域间基本公共服务大体均衡，贫困地区基本公共服务主要领域指标接近全国平均水平，广大群众享有基本公共服务的可及性显著提高。

　　--标准体系全面建立。国家基本公共服务清单基本建立，标准体系更加明确并实现动态调整，各领域建设类、管理类、服务类标准基本完善并有效实施。

　　--保障机制巩固健全。基本公共服务供给保障措施更加完善，基层服务基础进一步夯实，人才队伍不断壮大，供给模式创新提效，可持续发展的长效机制基本形成。

　　--制度规范基本成型。各领域制度规范衔接配套、基本完备，服务提供和享有有规可循、有责可究，基本公共服务依法治理水平明显提升。

　　“十三五”时期基本公共服务领域主要发展指标

指　　　　标	2015年	2020年	累　计

基本公共教育

九年义务教育巩固率（%）	93	95	-

义务教育基本均衡县（市、区）的比例（%）1	44.48	95	-

基本劳动就业创业

城镇新增就业人数（万人）2	-	-	＞5000

农民工职业技能培训（万人次）	-	-	4000

基本社会保险

基本养老保险参保率（%）3	82	90	-

基本医疗保险参保率（%）4	-	＞95	-

基本医疗卫生

孕产妇死亡率（1/10万）	20.1	18	-

婴儿死亡率（‰）	8.1	7.5	-

5岁以下儿童死亡率（‰）	10.7	9.5	-

基本社会服务

养老床位中护理型床位比例（%）	-	30	-

生活不能自理特困人员集中供养率（%）5	31.8	50	-

基本住房保障

城镇棚户区住房改造（万套）	-	-	2000

建档立卡贫困户、低保户、农村分散供养特困人员、贫困残疾人家庭等4类重点对象农村危房改造（万户）	-	-	585

基本公共文化体育

公共图书馆年流通人次（亿）	5.89	8	-

文化馆（站）年服务人次（亿）	5.07	8	-

广播、电视人口综合覆盖率（%）6	＞98	＞99	-

国民综合阅读率（%）7	79.6	81.6	-

经常参加体育锻炼人数（亿人）8	3.64	4.35	-

残疾人基本公共服务

困难残疾人生活补贴和重度残疾人护理补贴覆盖率（%）9	-	＞95	-

残疾人基本康复服务覆盖率（%）10	-	80	-

　　注：1.指通过省级评估、国家认定程序认定的义务教育均衡发展县（市、区）占全国所有县（市、区）的比例。

　　2.指城镇累计新就业人数减去累计自然减员人数。其中城镇累计新就业人数是指报告期内城镇累计新就业的城镇各类单位、私营企业和个体经济组织、社区公益性岗位就业人员和各种灵活形式就业人员的总和；累计自然减员人数是指报告期内因退休、伤亡等自然原因造成的城镇累计减少的就业人员数。

　　3.指按照国家有关法律和社会保险政策规定，实际参加基本养老保险的人数与法定应参加基本养老保险的人数之比。

　　4.指按照国家有关法律和社会保险政策规定，实际参加基本医疗保险的人数与法定应参加基本医疗保险的人数之比。

　　5.指在机构集中供养的生活不能自理特困人员与生活不能自理特困人员总数之比。

　　6.指在对象区内能接收到中央、省（区、市）、市（地、州）、县（市、区）广播、电视传输机构以无线、有线、卫星等方式传输的广播、电视节目信号的人口数占对象区总人口数的比重。

　　7.指全国每年有阅读行为（包括阅读书报刊物和数字出版物、手机媒体等各类读物）的人数与总人口数的比例。

　　8.指每周参加体育锻炼3次及以上、每次体育锻炼持续时间30分钟及以上、每次体育锻炼的运动强度达到中等及以上的人数。

　　9.指困难残疾人享受生活补贴和重度残疾人享受护理补贴的人数达到应享受补贴人数的比例。

　　10.指有康复需求的残疾儿童和持证残疾人接受康复评估、手术、药物、功能训练、辅具适配等基本康复服务的比例。

　第三章　国家基本公共服务制度

　第一节　制度框架

　　国家基本公共服务制度紧扣以人为本，围绕从出生到死亡各个阶段和不同领域，以涵盖教育、劳动就业创业、社会保险、医疗卫生、社会服务、住房保障、文化体育等领域的基本公共服务清单为核心，以促进城乡、区域、人群基本公共服务均等化为主线，以各领域重点任务、保障措施为依托，以统筹协调、财力保障、人才建设、多元供给、监督评估等五大实施机制为支撑，是政府保障全民基本生存发展需求的制度性安排。

　　国家基本公共服务制度框架

　第二节　服务清单

　　国家建立基本公共服务清单制，依据现行法律法规和相关政策确定基本公共服务主要领域，以及各领域具体服务项目和国家基本标准，向社会公布，作为政府履行职责和公民享有相应权利的依据。《“十三五”国家基本公共服务清单》（以下简称《清单》，详见附件1）包括公共教育、劳动就业创业、社会保险、医疗卫生、社会服务、住房保障、公共文化体育、残疾人服务等八个领域的81个项目。每个项目均明确服务对象、服务指导标准、支出责任、牵头负责单位等。其中，服务对象是指各项目所面向的受众人群；服务指导标准是指各项目的保障水平、覆盖范围、实现程度等；支出责任是指各项目的筹资主体及承担责任；牵头负责单位是指国家层面的主要负责单位，具体落实由地方各级人民政府及有关部门、单位按职责分工负责。

　　《清单》是“十三五”时期实现基本公共服务均等化的重要基础，各项目服务内容和标准要在规划期内落实到位。在本规划实施过程中，可结合经济社会发展状况，按程序对《清单》具体内容进行动态调整。

　第三节　实施机制

　　国家建立健全科学有效的基本公共服务实施机制，改善人财物等基础条件，以推动规划目标顺利实现，确保国家基本公共服务制度高效运转。

　　--统筹协调机制。加强中央和地方、政府和社会的互动合作，促进各级公共服务资源有效整合，形成实施合力。

　　--财力保障机制。拓宽资金来源，增强县级政府财政保障能力，稳定基本公共服务投入。

　　--人才建设机制。加强人才培养培训，强化激励约束，促进合理流动，相关政策重点向基层倾斜，不断提高服务能力和水平。

　　--多元供给机制。积极引导社会力量参与，推进政府购买服务，推广政府和社会资本合作（PPP）模式。

　　--监督评估机制。坚持目标导向和问题导向，完善信息统计收集和需求反馈机制，加强对本规划实施的动态跟踪监测，推动总结评估和督促检查。

　第四章　基本公共教育

　　国家完善基本公共教育制度，加快义务教育均衡发展，保障所有适龄儿童、青少年平等接受教育，不断提高国民基本文化素质。本领域服务项目共8项，具体包括：免费义务教育、农村义务教育学生营养改善、寄宿生生活补助、普惠性学前教育资助、中等职业教育国家助学金、中等职业教育免除学杂费、普通高中国家助学金、免除普通高中建档立卡等家庭经济困难学生学杂费。

　第一节　重点任务

　　--义务教育。建立城乡统一、重在农村的义务教育经费保障机制，加大对中西部和民族、边远、贫困地区的倾斜力度。统筹推进县域内城乡义务教育一体化改革发展，推进建设标准、教师编制标准、生均公用经费基准定额、基本装备配置标准统一和“两免一补”政策城乡全覆盖，基本实现县域校际资源均衡配置，扩大优质教育资源覆盖面，提高乡村学校和教学点办学水平。落实县域内义务教育公办学校校长、教师交流轮岗制度。保障符合条件的进城务工人员随迁子女在公办学校或通过政府购买服务在民办学校就学。加强国家通用语言文字教育基础薄弱地区双语教育。加强学校体育和美育教育。

　　--高中阶段教育。重点支持中西部贫困地区尤其是集中连片特困地区高中阶段教育发展，积极发展中等职业教育。逐步分类推进中等职业教育免除学杂费，率先从建档立卡等家庭经济困难学生（含非建档立卡的家庭经济困难残疾学生、农村低保家庭学生、农村特困救助供养学生）实施普通高中免除学杂费。

　　--普惠性学前教育。大力发展公办幼儿园，积极扶持民办幼儿园提供普惠性服务。扩大集中连片特困地区、少数民族地区学前教育资源。支持地方健全学前教育资助制度，资助普惠性幼儿园在园家庭经济困难儿童、孤儿和残疾儿童接受学前教育。

　　--继续教育。建立个人学习账号和学分累计制度，完善学分认定和转换办法，拓宽学分认定转换渠道，探索建立多种形式学习成果认定转换机制，促进各类学习资源开放共享，推动构建惠及全民的终身教育体系。

　第二节　保障措施

　　--义务教育学校标准化建设。以中西部贫困地区为重点，新建和改扩建校舍、运动场地、食堂（伙房）、厕所、饮水等设施条件，采购课桌凳、学生用床、图书、计算机等教学设施设备，全面改善贫困地区义务教育薄弱学校基本办学条件，逐步推进未达标城乡义务教育学校校舍、场所标准化。

　　--高中阶段教育设施建设。普及高中阶段教育，改善中西部贫困地区普通高中基本办学条件，逐步实现办学条件达到国家规定的基本标准。改善中等职业学校基本办学条件，重点支持中等职业学校通过校企合作方式加强实习实训设施建设，推动职业教育产教融合发展。

　　--学前教育行动计划。加强普惠性幼儿园建设，新建、改扩建一批公办幼儿园，积极扶持企事业单位办幼儿园、集体办幼儿园和民办幼儿园向社会提供普惠性学前教育服务，重点保障中西部农村适龄儿童和实施全面两孩政策新增适龄儿童入园需求。

　　--教师队伍建设。实施乡村教师支持计划。逐步扩大农村教师特岗计划实施规模。落实并完善集中连片特困地区和边远艰苦地区乡村教师生活补助政策。实施中西部中小学首席教师岗位计划，加大“国培计划”对中西部地区乡村教师校长培训的集中支持力度。加强乡村学校音体美等师资紧缺学科教师和民族地区双语教师培训。

　　--教育信息化建设。鼓励探索网络化教育新模式，对接线上线下教育资源，扩大优质教育资源覆盖面。加快推进“三通两平台”（即“宽带网络校校通、优质资源班班通、网络学习空间人人通”，教育资源公共服务平台、教育管理公共服务平台）建设与应用，继续提升农村中小学信息化水平，通过政府购买服务等方式支持国家级优质教育资源平台建设。建立个人学习账号和学分认证平台，为学习者提供学分认定服务。

　第五章　基本劳动就业创业

　　国家实施就业优先战略，大力推动大众创业、万众创新，鼓励以创业带动就业，健全覆盖城乡的公共就业创业服务体系，加强职业培训，维护职工和企业合法权益，构建和谐劳动关系，推动实现比较充分和更高质量的就业。本领域服务项目共10项，具体包括：基本公共就业服务、创业服务、就业援助、就业见习服务、大中城市联合招聘服务、职业技能培训和技能鉴定、“12333”人力资源和社会保障服务热线电话咨询、劳动关系协调、劳动人事争议调解仲裁、劳动保障监察。

　第一节　重点任务

　　--公共就业服务。全面落实就业政策法规咨询、信息发布、职业指导和职业介绍、就业登记和失业登记等公共就业服务制度，组织开展就业服务专项活动。加强对就业困难人员的就业援助，确保有就业能力的零就业家庭、低保家庭至少有一人就业。做好高校毕业生就业和农村劳动力转移就业，以及化解过剩产能过程中的职工安置工作。加快推进流动人员人事档案信息化服务。建立健全行业人力资源需求预测和就业状况定期发布制度，完善人力资源市场供求分析。

　　--创业服务。鼓励公共就业服务机构为创业者提供项目选择、开业指导、融资对接、跟踪扶持等服务。把创新创业课程纳入国民教育体系，建立健全衔接创业教育和创业实践的创业培训体系。深化行政审批制度改革和商事制度改革，推行市场主体登记注册便利化，减少政府对企业生产服务项目的行政许可和对正常经营活动的行政干预，落实降低企业负担的税费政策。落实创业担保贷款政策，提高就业重点群体和困难人员金融服务的可获得性。加快发展众创空间等创业服务载体，健全创业辅导制度。

　　--职业培训。大力开展就业技能培训、岗位技能提升培训和创业培训，开展贫困家庭子女、未升学初高中毕业生、农民工、失业人员和转岗职工、退役军人、残疾人免费接受职业培训行动，打通技能劳动者从初级工、中级工、高级工到技师、高级技师的职业发展通道。

　　--劳动关系协调和劳动权益保护。完善劳动用工制度，健全最低工资标准调整和工资支付保障长效机制，落实职工带薪年休假制度。加强劳动保障监察和劳动人事争议调解仲裁，推进劳动保障监察综合执法，建立劳动保障监察举报投诉案件省级联动处理机制，健全完善劳动人事争议多元处理机制，维护用人单位和劳动者合法权益。定期发布职业薪酬信息和重点行业人工成本信息。

　第二节　保障措施

　　--基层劳动就业和社会保障服务平台建设。充分依托现有条件和政府综合服务场所，完善县、乡镇两级劳动就业和社会保障服务设施设备，推进基层综合服务全覆盖，保障基层开展就业创业、社会保险经办等服务。

　　--职业技能公共实训基地建设。充分利用现有设施设备，结合地区实际，建设一批区域性大型公共实训基地、市级综合型公共实训基地和县级地方产业特色型公共实训基地。

　　--省、市级人力资源服务设施建设。充分依托现有条件和政府综合服务场所，完善省、市级人力资源综合服务设施，改善就业创业和人才服务、劳动关系协调、劳动人事争议调解仲裁、劳动保障监察等综合服务条件。

　　--信息服务平台建设。建设面向人人的公共就业创业服务平台，推进公共就业服务全程信息化，实现各类就业信息统一发布和信息监测。以“12333”电话咨询为重点，配备必要的服务场地和设施设备，健全咨询服务队伍和服务机制，为社会公众提供政策咨询、信息查询、信息公开、在线受理和投诉举报等服务。

　第六章　基本社会保险

　　国家构建全覆盖、保基本、多层次、可持续的社会保险制度，实施全民参保计划，保障公民在年老、疾病、工伤、失业、生育等情况下依法从国家和社会获得物质帮助。本领域服务项目共7项，具体包括：职工基本养老保险、城乡居民基本养老保险、职工基本医疗保险、生育保险、城乡居民基本医疗保险、失业保险、工伤保险。

　第一节　重点任务

　　--社会保险政策制度。继续实行统账结合的城镇职工基本养老保险制度，完善个人账户，健全激励约束机制，提高收付透明度，坚持精算平衡，推动实现职工基础养老金全国统筹。落实机关事业单位养老保险制度改革举措。推进实施城乡居民基本养老保险制度。健全基本医疗保险稳定可持续的筹资和报销比例调整机制，制定城乡居民医保政府补助三年规划，在提高政府补助标准的同时适当提高个人缴费比重，逐步将个人缴费与城乡居民家庭收入水平挂钩。完善医保缴费参保政策，改进个人账户，开展门诊费用统筹。实现基本医保基金中长期精算平衡，增强制度可持续性。改革医保支付方式，合理控制医疗费用，整合城乡居民医保政策和经办管理。全面实施城乡居民大病保险制度。将生育保险与基本医疗保险合并实施。探索建立长期护理保险制度，开展长期护理保险试点。继续完善预防、补偿、康复三位一体的工伤保险制度体系。推动医疗保险、失业保险、工伤保险逐步实现省级统筹。结合社会平均工资和物价变动等因素，合理确定相关社会保险待遇水平。

　　--社会保险关系转续。建立标准统一、全国联网的社会保障管理信息系统，完善并简化转续流程，推行网上认证、网上办理转续，力争实现全国范围内社会保险待遇异地领取、直接结算，方便参保职工、失业和退休人员流动就业、异地生活。

　第二节　保障措施

　　--社会保障卡工程。全面发行和应用社会保障卡，持卡人口覆盖率达到90%，实现社会保障一卡通，支持社会保障卡跨业务、跨地区、跨部门应用，建立社会保障卡应用平台和覆盖广泛的用卡终端环境，健全社会保障卡便民服务体系，完善社会保障卡规范管理和安全保障体系。

　　--省、市级社会保障服务设施建设。充分依托现有条件和政府综合服务场所，完善省、市级社会保障服务设施，推动改善社保经办等服务条件。

　　--全民社会保障信息化。建设部门和省级公共服务信息化平台，支持各类业务系统和各类服务渠道的统一接入、有序整合和统筹调度，推动电话、网站、移动应用、短信、自助服务一体机等多种渠道的协同应用，实现一个窗口对外、一条龙服务。开展网上社保办理、个人社保权益查询、跨地区医保结算等互联网应用。

　第七章　基本医疗卫生

　　国家建立健全覆盖城乡居民的基本医疗卫生制度，推进健康中国建设，坚持计划生育基本国策，以基层为重点，以改革创新为动力，预防为主、中西医并重，提高人民健康水平。本领域服务项目共20项，具体包括：居民健康档案、健康教育、预防接种、传染病及突发公共卫生事件报告和处理、儿童健康管理、孕产妇健康管理、老年人健康管理、慢性病患者管理、严重精神障碍患者管理、卫生计生监督协管、结核病患者健康管理、中医药健康管理、艾滋病病毒感染者和病人随访管理、社区艾滋病高危行为人群干预、免费孕前优生健康检查、基本药物制度、计划生育技术指导咨询、农村部分计划生育家庭奖励扶助、计划生育家庭特别扶助、食品药品安全保障。

　第一节　重点任务

　　--重大疾病防治和基本公共卫生服务。继续实施国家基本公共卫生服务项目和国家重大公共卫生服务项目。开展重大疾病和突发急性传染病联防联控，提高对传染病、慢性病、精神障碍、地方病、职业病和出生缺陷等的监测、预防和控制能力。加强突发公共事件紧急医学救援、突发公共卫生事件监测预警和应急处理。深入开展爱国卫生运动，继续推进卫生城镇创建工作，开展健康城市、健康村镇建设，实施全国城乡环境卫生整洁行动，加快农村改厕，农村卫生厕所普及率提高到85%。加强居民身心健康教育和自我健康管理，做好心理健康服务。

　　--医疗卫生服务。落实区域卫生规划和医疗机构设置规划，依据常住人口规模和服务半径等合理配置医疗卫生资源。深化基层医改，巩固完善基本药物制度，全面推进公立医院综合改革，推动形成基层首诊、双向转诊、急慢分治、上下联动的分级诊疗模式。完善中医医疗服务体系，发挥中医药特色优势，推动中医药传承与创新。

　　--妇幼健康和计划生育服务管理。实施全面两孩政策，改革完善计划生育服务管理，实施生育登记服务。开展孕前优生健康检查，加强高危孕产妇和新生儿健康管理。提高妇女常见病筛查率和早诊早治率，扩大农村妇女宫颈癌、乳腺癌项目检查覆盖范围。继续落实计划生育技术服务基本项目，将流动人口纳入城镇计划生育服务范围。加强出生人口性别比综合治理。完善农村部分计划生育家庭奖励扶助制度、计划生育家庭特别扶助制度，继续实施“少生快富”工程。

　　--食品药品安全。实施食品安全战略，完善法规制度，提高安全标准，全面落实企业主体责任，提高监督检查频次，扩大抽检监测覆盖面，实行全产业链可追溯管理。深化药品医疗器械审评审批制度改革，探索按照独立法人治理模式改革审评机构，推行药品经营企业分级分类管理。加大农村食品药品安全治理力度，完善对网络销售食品药品的监管。

　第二节　保障措施

　　--基层医疗卫生服务能力提升。在县级区域依据常住人口数，原则上办好1个县办综合医院和1个县办中医类医院（含中医、中西医结合、民族医等），每个乡镇（街道）办好1所标准化建设的乡镇卫生院（社区卫生服务中心），每个行政村办好1个村卫生室。优先支持832个国家扶贫开发工作重点县和集中连片特困地区县县级医院和基层医疗卫生机构建设，打造30分钟基层医疗服务圈，基层医疗卫生机构标准化达标率达到95%以上。

　　--疾病防治和基本公共卫生服务能力强化。加强卫生应急、疾病预防控制、精神卫生、血站、卫生计生监督能力建设。提高肿瘤、心脑血管疾病、呼吸系统疾病等疑难病症防治能力。支持肿瘤、心脑血管疾病、糖尿病、精神病、传染病、职业病、地方病等薄弱领域服务能力建设。

　　--妇幼健康服务保障。加强儿童医院和综合性医院儿科以及妇幼健康服务机构建设，合理增加产床。加快产科和儿科医师、助产士及护士人才培养，力争增加产科医生和助产士14万名。落实孕前优生健康检查，开展再生育技术服务。

　　--中医药传承创新。改善中医医院基础设施条件，支持中医重点学科和重点专科（专病）建设，加强中医临床研究基地和科研机构建设，鼓励基层医疗卫生机构开设中医综合服务区（中医馆），继续实施中医药传承与创新人才工程，实施中药民族药标准化行动。

　　--医疗卫生人才培养。加强住院医师规范化培训，力争到2020年经过规范化培训的住院医师数量达到50万人，每万人口全科医生数达到2名。继续实施助理全科医生培训、全科医生转岗培训和农村订单定向免费培养医学生政策，加强基层医务人员继续教育，完善城市医疗卫生人才对口支援农村制度。

　　--食品药品安全治理体系建设。完善食品安全协调工作机制，健全检验检测等技术支撑体系和信息化监管系统，建立食品药品职业化检查员队伍，实现各级监管队伍装备配备标准化。

　　--人口健康信息化。以全民健康保障信息化工程和健康中国云服务计划为基础，依托现有资源统筹建立人口健康信息平台。推进居民电子健康档案应用。积极利用移动互联网提供在线预约诊疗、健康咨询、检查检验报告查询等服务，提高重大疾病和突发公共卫生事件防控能力。完善中西部地区县级医院电子病历等信息系统功能，加强县级医院与对口三级医院、县级医院与基层医疗卫生机构之间的远程诊疗信息系统建设，健全基于互联网、大数据技术的分级诊疗信息系统。

　第八章　基本社会服务

　　国家建立完善基本社会服务制度，为城乡居民提供相应的物质和服务等兜底帮扶，重点保障特定人群和困难群体的基本生存权与平等参与社会发展的权利。本领域服务项目共13项，具体包括：最低生活保障、特困人员救助供养、医疗救助、临时救助、受灾人员救助、法律援助、老年人福利补贴、困境儿童保障、农村留守儿童关爱保护、基本殡葬服务、优待抚恤、退役军人安置、重点优抚对象集中供养。

　第一节　重点任务

　　--社会救助。推进城乡低保统筹发展，健全低保对象认定办法，建立低保标准动态调整机制，确保农村低保标准逐步达到国家扶贫标准。完善特困人员认定条件，合理确定救助供养标准，适度提高救助供养水平。合理界定医疗救助对象，健全疾病应急救助制度，全面开展重特大疾病医疗救助工作，加强医疗救助与基本医疗保险、大病保险和其他救助制度的衔接。全面、高效实施临时救助制度。降低法律援助门槛，扩大法律援助范围。

　　--社会福利。全面建立针对经济困难高龄、失能老年人的补贴制度，并做好与长期护理保险的衔接。提高城乡社区卫生服务机构为老年人提供医疗保健服务的能力，加快社区居家养老信息网络和服务能力建设，推进医养结合发展。进一步完善孤儿基本生活保障制度，做好困境儿童保障工作，统筹推进未成年人社会保护试点和农村留守儿童关爱保护。全面推进精神障碍患者社区康复服务。

　　--社会事务。建立和完善公民婚姻信息数据库，探索开展异地办理婚姻登记工作。完善儿童被收养前寻亲公告程序，全面建立收养能力评估制度。推进基本殡葬公共服务，巩固提高遗体火化率，推行火葬区骨灰和土葬改革区遗体规范、集中节地生态安葬。做好第二次全国地名普查，健全地名管理法规标准，加强地名文化保护，开展多种形式的地名信息化服务。

　　--优抚安置。全面落实优抚安置各项制度政策，提升对复员退伍军人、军休人员的优抚安置和服务保障能力。完善优抚政策和优抚对象抚恤优待标准调整机制。将优抚安置对象优先纳入社区、养老、医疗卫生等服务体系，探索建立优抚安置对象社会化服务平台。

　第二节　保障措施

　　--社会救助经办服务体系建设。充分依托现有条件和政府综合服务场所，推动乡镇人民政府和街道办事处设置社会救助经办平台，加强社会救助管理信息系统与居民家庭经济状况核对系统的整合、集成，提升基层社会救助经办服务能力。

　　--公共法律服务体系建设。加强法律援助综合服务平台和便民窗口、法律服务中心（站、工作室）、“12348”法律服务热线等基础设施建设，改善服务条件。加强基层普法阵地、人民调解组织、司法鉴定机构建设，健全服务网络。

　　--养老服务体系建设。支持主要面向失能、半失能老年人的老年养护院，医养结合设施和社区老人日间照料中心，荣誉军人休养院、光荣院，农村特困人员救助供养服务机构等服务设施建设，增加护理型床位和设施设备。推进无障碍通道、老年人专用服务设施、旧楼加建电梯建设，以及适老化路牌标识、适老化照明改造。积极开展养老护理人员培养培训。搭建养老信息服务网络平台，推广应用便携式体检、紧急呼叫监控等设备。

　　--社会福利服务设施建设。结合地区实际，建设一批县级儿童福利设施。依托现有设施资源，试点建设县级未成年人保护设施。支持尚无精神病人福利设施的地市建设一所精神病人福利设施，为特殊困难精神障碍患者提供集中养护服务。

　　--殡葬服务设施建设。在火葬区尚无殡仪馆的县（市、区）新建殡仪馆，对已达危房标准、设施设备陈旧的殡仪馆进行改造或改扩建。更新改造已达到强制报废年限或不符合国家环境保护标准的火化炉。试点建设县（市、区）公益性骨灰安放设施。

　　--自然灾害救助物资储备体系建设。进一步优化中央救灾物资储备库布局，设区的市级以上人民政府和自然灾害多发、易发地区的县级人民政府应当根据自然灾害特点、居民人口数量和分布等情况，按照布局合理、规模适度的原则，设立救灾物资储备库（点），并视情在多灾易灾乡镇（街道）和城乡社区设置救灾物资储备室。

　　--社会工作者队伍建设。实施社会工作专业人才服务贫困地区计划、农村留守人员社会保护计划、城镇流动人口社会融入计划、特殊群体社会关爱计划，推进社会工作者专业化、职业化，力争到2020年社会工作专业人才总规模达145万人。

　第九章　基本住房保障

　　国家建立健全基本住房保障制度，加大保障性安居工程建设力度，加快解决城镇居民基本住房问题和农村困难群众住房安全问题，更好保障住有所居。本领域服务项目共3项，具体包括：公共租赁住房、城镇棚户区住房改造、农村危房改造。

　第一节　重点任务

　　--公共租赁住房。转变公租房保障方式，实行实物保障与租赁补贴并举，推进公租房货币化。支持公租房保障对象通过市场租房，政府对符合条件的家庭给予租赁补贴。完善租赁补贴制度，结合市场租金水平和保障对象实际情况，合理确定租赁补贴标准。在城镇稳定就业的外来务工人员、新就业大学生和青年医生、青年教师等专业技术人员，符合当地城镇居民公租房准入条件的，应纳入公租房保障范围。提高公租房运营保障能力，健全准入退出管理机制。

　　--城镇棚户区住房改造。围绕实现约1亿人居住的城镇棚户区、城中村和危房改造目标，实施棚户区改造行动计划和城镇旧房改造工程，基本完成城镇棚户区和危房改造任务。将棚户区改造与城市更新、产业转型升级更好结合起来，加快推进集中成片棚户区和城中村改造，有序推进旧住宅小区综合整治、危旧住房和非成套住房改造，棚户区改造政策覆盖全国重点镇。完善配套基础设施，加强工程质量监管。

　　--农村危房改造。合理确定农村危房改造补助对象和标准，优先帮助住房最危险、经济最贫困农户解决最基本的住房安全问题。加快推进贫困地区危房改造，按照精准扶贫、精准脱贫要求，重点解决建档立卡贫困户、低保户、农村分散供养特困人员、贫困残疾人家庭的基本住房安全问题。

　第二节　保障措施

　　--保障必要用地需求。在土地利用年度计划中根据保障性住房建设需要，单独列出，做到应保尽保。依法收回的闲置土地、具备净地出让条件的储备土地和农用地转用计划指标，应优先保证保障性住房用地需求。

　　--实施财税优惠政策。统筹运用政府财力，加大对基本住房保障的支持力度。继续落实好城镇保障性安居工程建设和运营管理涉及的行政事业性收费、政府性基金（含土地出让收入）以及相关税收减免政策。土地出让收益用于保障性安居工程的比例不低于10%。

　　--加大融资支持力度。支持符合条件的企业发行债券融资，用于保障性安居工程建设。进一步发挥开发性、政策性金融机构作用，加大对棚户区改造项目的信贷支持力度。鼓励商业银行在风险可控、商业可持续的前提下，开发适合住房租赁业务发展需要的信贷产品。

　　--合理确定住房价格。依据当地经济社会发展水平、保障对象的承受能力以及建设成本等因素，合理制定、调整保障性住房价格或租金标准。

　第十章　基本公共文化体育

　　国家构建现代公共文化服务体系和全民健身公共服务体系，促进基本公共文化服务和全民健身基本公共服务标准化、均等化，更好地满足人民群众精神文化需求和体育健身需求，提高全民文化素质和身体素质。本领域服务项目共10项，具体包括：公共文化设施免费开放、送地方戏、收听广播、观看电视、观赏电影、读书看报、少数民族文化服务、参观文化遗产、公共体育场馆开放、全民健身服务。

　第一节　重点任务

　　--公共文化。落实国家基本公共文化服务指导标准和地方实施标准。深化公益性文化事业单位改革，积极搭建公益性文化活动平台，以群众需求为导向，推行“菜单式”、“订单式”公共文化服务。加大政府向社会力量购买公共文化服务力度。深入推进公共图书馆、博物馆、美术馆、文化馆和综合文化站免费开放工作。以县级文化馆、图书馆为中心推进总分馆制，实现农村、城市社区公共文化服务资源整合和互联互通。加强文化遗产保护。

　　--广播影视。采用地面无线、直播卫星和有线网络等方式，推动数字广播电视基本实现全覆盖、户户通。进一步改善农村电影放映条件。努力增加贴近基层群众需要的服务性广播电视栏目节目。

　　--新闻出版。推动全民阅读，加强残疾人等特殊群体的基本阅读权益保障。扶持实体书店发展，加快推进实体书店或各类图书代销代购网点覆盖全国所有乡镇。完善农家书屋出版物补充更新工作。加强“三农”出版物出版发行。推动少数民族语言文字及双语出版物出版发行、数字化传播和少数民族语言文字作品创作。

　　--群众体育。实施全民健身计划，组织实施国民体质监测，推行《国家体育锻炼标准》，开展全民健身活动，实行科学健身指导。推动公共体育场馆向社会免费或低收费开放。全面实施青少年体育活动促进计划，培养青少年体育爱好和运动技能，推广普及足球、篮球、排球和冰雪运动等。

　第二节　保障措施

　　--公共文化服务体系建设。推动各地区进一步完善图书馆、文化馆（站）、博物馆等基本公共文化服务设施。在乡镇（街道）和村（社区）统筹建设集宣传文化、党员教育、科学普及、普法教育、体育健身等功能于一体的综合性文化服务中心。为集中连片特困地区和西藏、四省藏区、新疆南疆四地州以及国家扶贫开发工作重点县、新疆生产建设兵团边境团场和南疆困难团场每个县级文化馆配备一辆流动文化车，为村文化活动室购置基本公共文化服务设备。

　　--广播影视服务体系建设。加强广播电视数字化覆盖、广播电视无线发射台站、全国有线电视网络互联互通平台、国家和地方应急广播体系、基层广播电视播出机构制播能力、广播电视和视听新媒体监管平台等建设，支持直播卫星平台扩容。实施农村电影放映工程，继续巩固“一行政村一月放映一场电影”成果。加强少数民族语言广播影视节目译制、制作、播出和传输覆盖能力建设。

　　--新闻出版服务体系建设。举办“书香中国”系列活动，充分利用现有设施，统筹建设社区阅读中心、数字农家书屋、公共数字阅读终端等设施。合理规划建设农村和中小城市出版物发行网点，建设城乡阅报栏（屏），支持革命老区、民族地区、边疆地区、贫困地区公共阅读设施建设。实施少数民族新闻出版东风工程、盲文出版工程、儿童阅读书报发放计划、市民阅读发放计划。

　　--遗产保护服务体系建设。重点支持全国重点文物保护单位、国家历史文化名城、国家级非物质文化遗产、国家级风景名胜区、国家森林公园、国家地质公园等文化和自然遗产保护利用设施建设。

　　--公共体育服务设施建设。重点支持足球场地设施、中小型全民健身中心、县级体育场、农民体育健身工程、社区多功能运动场、冰雪运动设施、科学健身指导服务平台等建设。充分利用体育中心、公园绿地、闲置厂房、校舍操场、社区空置场所等，拓展公共体育设施场所。

　　--数字文化服务平台建设。推动全国文化信息资源共享、数字图书馆博物馆建设等公共数字文化工程建设。提高公共文化大数据采集、存储和分析处理能力。科学规划公共数字文化资源，建设分布式资源库群，实施“互联网＋中华文明”行动计划，鼓励各地区挖掘整合中华优秀文化资源，开发特色数字文化产品。

　第十一章　残疾人基本公共服务

　　国家提供适合残疾人特殊需求的基本公共服务，为残疾人平等参与社会发展创造便利化条件和友好型环境，让残疾人安居乐业、衣食无忧，生活得更加殷实、更加幸福、更有尊严。本领域服务项目共10项，具体包括：困难残疾人生活补贴和重度残疾人护理补贴、无业重度残疾人最低生活保障、残疾人基本社会保险个人缴费资助和保险待遇、残疾人基本住房保障、残疾人托养服务、残疾人康复、残疾人教育、残疾人职业培训和就业服务、残疾人文化体育、无障碍环境支持。

　第一节　重点任务

　　--残疾人基本生活。全面落实困难残疾人生活补贴和重度残疾人护理补贴制度。生活困难、靠家庭供养且无法单独立户的成年无业重度残疾人，经个人申请，可按照单人户纳入最低生活保障范围。对获得最低生活保障后仍有困难的重度残疾人采取必要措施给予生活保障。完成农村贫困残疾人家庭存量危房改造。

　　--残疾人就业创业和社保服务。为有劳动能力和就业意愿的城乡残疾人免费提供就业创业服务，按规定提供免费职业培训。落实好针对就业困难残疾人的各项就业援助和扶持政策，为智力、精神和重度肢体残疾人提供辅助性、支持性就业服务等。落实贫困和重度残疾人参加社会保险个人缴费资助政策，完善重度残疾人医疗报销制度，做好重度残疾人就医费用结算服务。

　　--残疾人康复、教育、文体和无障碍服务。继续实施残疾儿童抢救性康复、贫困残疾人辅助器具适配、防盲治盲、防聋治聋等重点康复项目，加强残疾人健康管理和社区康复。积极推进为家庭经济困难的残疾儿童、青少年提供包括义务教育和高中阶段教育在内的12年免费教育。加强国家通用手语、通用盲文的规范与推广。推动公共文化体育场所设施免费或优惠向残疾人开放，为视力、听力残疾人等提供特需文化服务。加快推进公共场所和设施的无障碍改造。

　第二节　保障措施

　　--残疾人服务体系建设。支持各地建设一批专业化残疾人康复设施、托养设施和综合服务设施，配备基本服务设备，推动形成功能完善、网络健全的残疾人专业康复和托养服务体系。

　　--县域残疾人综合服务能力提升。强化县级残疾人康复、托养、职业培训、辅助器具适配、文化体育等服务能力，充分发挥基层公共服务设施助残功能，推动形成县（市、区）、乡（镇）、村（居）三级联动互补的残疾人基层服务网络。

　　--特殊教育基础能力提升。依托现有特教学校构建特殊教育资源中心，提升特殊教育普及水平、保障条件和教育质量。完善特殊教育体系，积极创造条件保障完成义务教育且有意愿的残疾学生有机会接受适宜的中等职业教育。

　　--残疾人服务专业人才培养。建设康复大学，提升高等院校特殊教育专业办学水平，推动师范院校开设特殊教育课程。加快培养残疾人康复、托养、特殊教育、护理照料、就业服务、社会工作等方面的人才队伍。

　　--残疾人服务信息化。完善残疾人人口基础信息和基本服务需求信息数据管理系统。依托中国残疾人服务网，搭建残疾人就业创业网络服务平台。加快推进智能化残疾人证试点。鼓励支持服务残疾人的电子产品、移动应用软件等开发应用。

　第十二章　促进均等共享

　　以贫困地区和贫困人口为重点，着力扩大覆盖范围、补齐短板、缩小差距，不断提高城乡、区域、人群之间基本公共服务均等化程度。

　第一节　推动基本公共服务全覆盖

　　--开展贫困地区脱贫攻坚。加大革命老区、民族地区、边疆地区、集中连片特困地区脱贫攻坚力度，保障贫困人口享有义务教育、医疗卫生、文化体育、住房安全等基本公共服务，推动贫困地区基本公共服务主要领域指标接近全国平均水平。深入开展教育扶贫、健康扶贫、文化扶贫。在易地扶贫搬迁、整村推进、就业促进等工作中，按照精准扶贫、精准脱贫的要求，确保基本公共服务不留缺口。推动地区对口帮扶，加大基本公共服务资金、项目和人才支援力度。

　　--重点帮扶特殊困难人群。对农村留守人员、困境儿童和残疾人进行全面摸底排查，建立翔实完备、动态更新的信息台账。逐步完善救助管理机构、福利机构场所设施条件，满足农村留守儿童临时监护照料需要。在外出就业较为集中的农村地区，充分利用布局调整后闲置资源开展托老、托幼等关爱服务。健全孤儿、弃婴、法定抚养人无力抚养儿童、低收入家庭重病重残等困境儿童的福利保障体系。对低保家庭中的老年人、未成年人、重度残疾人等重点救助对象，提高救助水平，保障基本生活。

　　--促进城镇常住人口全覆盖。深化户籍制度改革，推动有能力在城镇稳定就业和生活的农业转移人口举家进城落户。推进居住证制度覆盖全部未落户城镇常住人口，加大对农业转移人口市民化的财政支持力度并建立动态调整机制，保障居住证持有人在居住地享有教育、就业、卫生等领域的基本公共服务。为农民工提供新市民培训服务，提高农民工综合素质和融入城市的能力。

　第二节　促进城乡区域均等化

　　--缩小城乡服务差距。加快义务教育、社会保障、公共卫生、劳动就业等制度城乡一体设计、一体实施。重点以县（市、区）为单位，有步骤、分阶段推动规划、政策、投入、项目等同城化管理，统筹设施建设和人员安排，推动城乡服务内容和标准统一衔接。把社会事业发展重点放在农村和接纳农业转移人口较多的城镇，补齐农村和特大镇基本公共服务短板。鼓励和引导城镇公共服务资源向农村延伸，促进城市优质资源向农村辐射。

　　--提高区域服务均等化水平。强化省级人民政府统筹职能，加大对省域内基本公共服务薄弱地区扶持力度，通过完善事权划分、规范转移支付等措施，逐步缩小县域间、地市间服务差距。强化跨区域统筹合作，促进服务项目和标准水平衔接。着力推进京津冀地区、长江经济带等重点区域基本公共服务均等化，形成可复制、可推广的经验。

　　--夯实基层服务基础。整合相关资源，持续改善基层各类公共服务设施条件。依托政府综合服务大厅完善相关经办服务设施，推动基层综合公共服务平台统筹发展和共建共享。简化基层办事环节和手续，优化服务流程，明确办理时限，推行一站式办理、上门办理、预约办理等服务方式。在山区、草原等地广人稀、居住分散地区，配备必要的教学点，开展卫生巡诊等上门服务。

　第十三章　创新服务供给

　　紧扣增进民生福祉，加快推进社会事业改革，吸引社会力量参与，扩大基本公共服务有效供给，提高服务质量和水平。

　第一节　培育多元供给主体

　　--加快事业单位分类改革。理顺政府与事业单位在基本公共服务供给中的关系，强化提供基本公共服务事业单位的公益属性，推动去行政化和去营利化，逐步将有条件的事业单位转为企业或社会组织。进一步落实事业单位法人自主权，深化人事、收入分配等配套制度改革，确保依法决策、独立自主开展活动并承担责任。

　　--积极引导社会力量参与。进一步规范和公开基本公共服务机构设立的基本标准、审批程序，严控审批时限，鼓励有条件的地方采取招标等方式确定举办或运营主体。积极推动基本公共服务领域民办非营利性机构享受与同行业公办机构同等待遇。

　　--大力发展社会组织。深化社会组织登记管理制度改革，落实税收优惠政策。加强社会组织孵化培育和人才扶持，采取人员培训、项目指导、公益创投等多种途径和方式，提升社会组织承接政府购买服务能力。采取降低准入门槛、加强分类指导和业务指导等办法，大力培育发展社区社会组织，支持其承接基层基本公共服务和政府委托事项。

　第二节　推动供给方式多元化

　　--推进政府购买公共服务。能由政府购买服务提供的，政府不再直接承办，交由具备条件、信誉良好的社会组织、机构、事业单位和企业等承担。制定实施政府购买公共服务指导性目录，确定政府购买公共服务的种类、性质和内容，规范项目遴选、信息发布、组织购买、项目监管、绩效评价等流程，加强政府购买公共服务的财政预算管理。

　　--加强政府和社会资本合作。能由政府和社会资本合作提供的，广泛吸引社会资本参与。政府通过投资补助、基金注资等多种方式，优先支持PPP项目。在实践证明有效的领域，推行通过公开招标、邀请招标、竞争性磋商、竞争性谈判等多种方式，公平选择具有相应管理经验、专业能力、融资实力以及信用状况良好的社会资本作为合作伙伴。

　　--鼓励发展志愿和慈善服务。广泛动员志愿服务组织与志愿者参与基本公共服务提供，定期发布志愿服务项目需求和岗位信息，建立健全志愿服务记录制度，完善激励保障措施。发挥慈善组织、专业社会工作服务机构在基本公共服务提供中的重要补充作用，落实慈善捐赠的相关优惠政策。

　　--发展“互联网＋”益民服务。加快互联网与政府公共服务体系的深度融合，推动公共数据资源开放，促进公共服务创新供给和服务资源整合，构建面向公众的一体化在线公共服务体系。推动具备条件的服务事项实行网上受理、网上办理、网上反馈、实时查询，对暂不具备条件的事项提供全程在线咨询服务。积极应用大数据理念、技术和资源，及时了解公众服务需求和实际感受，为政府决策和监管提供支持。

　　--扩大开放交流合作。鼓励通过合资、合作等方式，支持合作办医，共建养老和残疾人托养机构。加强公共教育、公共文化体育等领域对外交流与合作。借鉴国际先进管理和服务经验，提升基本公共服务供给质量和水平。

　第十四章　强化资源保障

　　优化资源配置，加强财力保障，加大重大工程项目、服务管理人才和规划用地等投入力度，为促进基本公共服务均等化提供支撑。

　第一节　提升财政保障能力

　　--加大财政投入力度。稳定基本公共服务投入，明确保障措施和《清单》项目支出责任，确保服务项目及标准落实到位。中央和地方各级财政要为提高贫困地区基本公共服务水平提供必要支持。加大地方政府债券对基本公共服务保障的支持力度。

　　--优化转移支付结构。合理划分中央和地方财政事权与支出责任，适度加强中央政府承担基本公共服务的职责和能力。推进转移支付制度改革，增加一般性转移支付规模和比例，重点增加对老少边穷地区的转移支付，缩小地区间财力差距，提高县级财政保障能力，引导地方将一般性转移支付资金投入到民生等重点领域。对新疆维吾尔自治区、新疆生产建设兵团、西藏自治区、四省藏区、革命老区、集中连片特困地区的民生保障和改善、基础设施建设、基层政权和社会管理能力建设等项目，中央预算内投资给予倾斜支持。

　　--提高资金使用效率。清理、整合、规范专项转移支付，完善资金管理办法，提高项目管理水平。简化财政管理层级，扩大省直管县财政管理体制改革覆盖面，加大省级人民政府转移支付对省域内基本公共服务财力差距的调节力度。统筹安排、合理使用、规范管理各类公共服务投入资金。对医院、学校、保障性住房等建筑质量实行单位负责人和项目负责人终身负责制。

　第二节　加强人才队伍建设

　　--加强人才培养培训。支持高等院校和中等职业学校开设相关学科专业，扩大专业服务和管理人才培养规模。健全从业人员继续教育制度，强化定岗、定向培养，完善远程教育培训。建立政府、社会、用人单位和个人相结合的投入机制，对参加相关职业培训和职业技能鉴定的人员，按规定给予补贴。探索公办与非公办公共服务机构在技术和人才等方面的合作机制，对非公办机构的人才培养、培训和进修等给予支持。

　　--促进人才合理流动。实施东部带西部、城市带农村的人才对口支持政策，引导公共服务和管理人才向中西部地区和基层流动。深化公办机构人事制度改革，健全公开招聘和竞争上岗制度，推动服务人员保障社会化管理，逐步由身份管理向岗位管理转变。

　　--提升基层人员能力。完善基层人员工资待遇、职称评定、医疗保险及养老保障等激励政策。推进基层公共服务队伍轮训，实施高校毕业生基层培养计划，继续做好“三支一扶”计划、西部志愿者计划、大学生村官计划、农村教师特岗计划、全科医生特岗计划、社会工作专业人才队伍建设等工作。鼓励通过优化编制资源配置、积极推进政府购买服务等方式，保障基层服务力量。

　第三节　完善配套政策体系

　　--加强规划布局和用地保障。综合服务半径、服务人口、资源承载能力等因素，对城乡公共服务设施进行统筹布局。结合新型城镇化和人口发展趋势，对土地供给进行前瞻规划，优先保障基本公共服务建设用地。新建居住区要按相关规定，完善教育、卫生、文化体育、养老托幼、社区服务等配套设施，并在合理服务半径内尽量集中安排。

　　--建立健全服务标准体系。各行业主管部门会同国务院标准化行政主管部门等，分别制定实施基本公共服务各领域设施建设、设备配置、人员配备、经费投入、服务规范和流程等具体标准，推动城乡、区域之间标准衔接。推进基本公共服务标准化工程建设，在有条件的地区开展公共服务标准化试点。

　　--强化社会信用体系支撑。增强全民诚信意识，健全个人信用档案。加强公共服务行业自律和社会监督，将公共服务机构、从业人员、服务对象诚信情况记入信用记录，纳入全国信用信息共享平台，对严重失信主体采取失信惩戒或依法强制退出等措施。

　第十五章　推进规划实施和监督评估

　　按照长效可行、分工明晰、统筹有力、协调有序的要求，扎实推进规划实施和监督评估，促进政策和项目落地。

　第一节　明确责任分工

　　--国务院各有关部门要按照职责分工，做好行业发展规划、专项建设规划与本规划的衔接，明确工作责任和进度安排，推动各领域重点任务、保障措施和《清单》项目有效落实。要加强部门间统筹协调，共同研究推动解决基本公共服务均等化工作中跨部门、跨行业、跨区域及政策创新等重大问题。

　　--省级人民政府要强化主体责任，以本规划为指导，结合实际制定推进本地区基本公共服务均等化规划、行动计划或基本公共服务清单，科学确定服务范围和项目内容，分年足额落实财政投入，切实促进省域内基本公共服务均等化。

　　--市、县级人民政府负责推进落实国家和省级人民政府确定的基本公共服务清单及相关政策措施，制定办事指南，明确责任单位，优化服务流程，提高质量效率，保证清单项目落实到位，并及时向上级政府和有关部门报告进展情况。

　第二节　加强监督问责

　　--国家发展改革委要会同国家统计局等有关部门，建立健全基本公共服务综合评估指标体系，推进基本公共服务基础信息库建设，开展年度统计监测。适时组织开展本规划实施情况中期评估，重大情况及时向国务院报告。

　　--国务院各有关部门、地方各级人民政府要建立政府主导与社会参与的良性互动机制，推动政务公开和政府信息公开，拓展公众参与渠道，做好舆情监测预警和应对，定期开展基本公共服务需求分析和社会满意度调查，及时妥善回应社会关切。

　　--地方各级人民政府要加强绩效评价和监督问责，强化过程监管，把本规划落实情况纳入绩效考核。要依法接受同级人大及其常委会的监督，自觉接受人民政协的民主监督，接受社会和人民群众监督。

　　附件：1.“十三五”国家基本公共服务清单

　　2.重点任务分工方案

　　附件1

　　“十三五”国家基本公共服务清单

序号	服务项目	服务对象	服务指导标准	支出责任	牵头负责单位

一、基本公共教育

1	免费义务教育	义务教育学生	对城乡义务教育学生免除学杂费，免费提供教科书；统一城乡义务教育学校生均公用经费基准定额。	中央和地方财政按比例分担。	财政部、教育部

2	农村义务教育学生营养改善	贫困地区农村义务教育学生	在集中连片特困地区开展国家试点，中央财政为试点地区学生提供每生每年800元的营养膳食补助，鼓励各地因地制宜开展地方试点。	国家试点县学生营养膳食补助所需资金由中央财政承担；地方试点县学生营养膳食补助所需资金由地方财政承担，中央财政给予奖励性补助。	教育部、财政部

3	寄宿生生活补助	义务教育家庭经济困难寄宿学生	小学生每生每年1000元，初中生每生每年1250元。	中央和地方财政按5∶5比例共同分担。	财政部、教育部

4	普惠性学前教育资助	经县级以上教育行政部门审批设立的普惠性幼儿园在园家庭经济困难儿童、孤儿和残疾儿童	减免保育教育费，补助伙食费，具体资助方式和资助标准由省级人民政府结合本地实际自行制定。	地方人民政府负责，中央财政予以奖补。按照“地方先行，中央补助”的原则开展相关工作。	财政部、教育部

5	中等职业教育国家助学金	中等职业学校全日制正式学籍一、二年级在校涉农专业学生和非涉农专业家庭经济困难学生；六盘山区等11个集中连片特困地区和西藏、四省藏区、新疆南疆四地州中等职业学校农村（不含县城）学生	国家助学金每生每年2000元，中央财政按区域确定家庭经济困难学生比例，西部地区按在校学生的20%确定，中部地区按在校学生的15%确定，东部地区按在校学生的10%确定。	中央和地方财政按比例分担：西部地区（不分生源地）以及中部、东部地区（生源地为西部的），中央与地方分担比例为8∶2；对中部地区（生源地不是西部的）以及东部地区生源地为中部的，中央与地方分担比例为6∶4；东部地区（生源地不是西部、中部的）分担比例分省（市）确定。	财政部、教育部、人力资源社会保障部

6	中等职业教育免除学杂费	公办中等职业学校全日制正式学籍一、二、三年级在校生中所有农村（含县镇）学生，城市涉农专业学生和家庭经济困难学生（艺术类相关表演专业学生除外），符合条件的民办职业学校学生	按各省（区、市）人民政府及其价格、财政主管部门确定的学费标准免除学杂费。公办中等职业学校，中央财政统一按平均每生每年2000元标准，与地方按比例分担免除学杂费补助资金。符合条件的民办职业学校学生参照当地同类型、同专业公办学校免除学杂费标准予以补助。	中央和地方财政按比例分担：西部地区（不分生源地）以及中部、东部地区（生源地为西部的），中央与地方分担比例为8∶2；对中部地区（生源地不是西部的）以及东部地区生源地为中部的，中央与地方分担比例为6∶4；东部地区（生源地不是西部、中部的）分担比例分省（市）确定。	财政部、教育部、人力资源社会保障部

7	普通高中国家助学金	普通高中在校生中的家庭经济困难学生	国家助学金平均资助标准为每生每年2000元，具体标准由各地结合实际分档确定。	中央和地方财政按比例分担：西部地区中央与地方分担比例为8∶2；中部地区分担比例为6∶4；东部地区除直辖市外，按照财力状况分省确定。	财政部、教育部

8	免除普通高中建档立卡等家庭经济困难学生学杂费	公办普通高中建档立卡等家庭经济困难在校学生（含非建档立卡的家庭经济困难残疾学生、农村低保家庭学生、农村特困救助供养学生），符合条件的民办普通高中学生	按各省（区、市）人民政府及其价格、财政主管部门确定的学费标准免除学杂费（不含住宿费）。中央财政逐省（区、市）核定免学杂费财政补助标准。符合条件的民办学校学生参照当地同类型公办学校免除学杂费标准予以补助。	中央和地方财政按比例分担：西部地区中央与地方分担比例为8∶2；中部地区分担比例为6∶4；东部地区除直辖市外，按照财力状况分省确定。	财政部、教育部

二、基本劳动就业创业

9	基本公共就业服务	有就业需求的劳动年龄人口	提供就业政策法规咨询、职业供求信息、市场工资指导价位信息和职业培训信息、职业指导和职业介绍、就业登记和失业登记、流动人员人事档案管理等服务。	国务院有关部门所属人才中介服务机构开展流动人员人事档案管理所需经费由中央财政予以补助，其余由地方人民政府负责。	人力资源社会保障部

10	创业服务	有创业需求的劳动者	提供项目选择、开业指导、融资对接、岗位信息等服务，对符合政策规定的创业者提供创业担保贷款扶持。	地方人民政府负责。	人力资源社会保障部、财政部、人民银行

11	就业援助	零就业家庭和符合条件的就业困难人员	提供政策咨询、职业指导、岗位信息等服务，使城镇有就业能力的零就业家庭至少一人就业。	地方人民政府负责。	人力资源社会保障部

12	就业见习服务	离校一年内未就业高校毕业生	组织有意愿的离校未就业毕业生参加就业见习；指导见习单位和见习人员签订见习协议，安排带教老师，为见习人员办理人身意外保险；见习单位和地方人民政府为见习人员提供基本生活补助。对见习期满留用率达到50%以上的见习单位，适当提高见习补贴标准。	见习人员基本生活补助所需资金由见习单位和地方人民政府分担。	人力资源社会保障部、财政部

13	大中城市联合招聘服务	有求职愿望的高校毕业生和青年人才以及有招聘需求的各类用人单位	提供大中城市联动、线上线下融合的招聘服务，方便服务对象登录用人单位需求库和求职简历库；提供职业能力测试和评估、简历（岗位）筛查和需求分析、预就业创业体验、双向定制推荐岗位（人才）信息、就业创业指导、实用基础课程培训等就业服务。	地方人民政府负责。	人力资源社会保障部

14	职业技能培训和技能鉴定	城乡各类有就业创业、提升岗位技能要求和培训愿望的劳动者	贫困家庭子女、毕业年度高校毕业生、城乡未继续升学的应届初高中毕业生、农村转移就业劳动者、城镇登记失业人员，以及符合条件的企业在职职工可按规定享受职业培训补贴；按规定给予参加劳动预备制培训的农村学员和城市低保家庭学员一定生活费补贴；符合条件人员享受职业技能鉴定补贴。	地方人民政府负责，国家给予适当补助。	人力资源社会保障部、财政部

15	“12333”人力资源和社会保障服务热线电话咨询	所有单位和个人	提供就业、社会保障、劳动关系、人事制度、人才建设、工资收入分配等方面的政策咨询及信息查询服务。人工服务为5×8小时，自助语音服务为7×24小时，综合接通率达到80%以上。	地方人民政府负责。	人力资源社会保障部

16	劳动关系协调	用人单位和与之建立劳动关系的劳动者	提供劳动关系政策咨询、劳动用工指导、获得劳动合同和集体合同示范文本、劳动纠纷调解、集体协商指导等服务，推动企业劳动合同签订率达到90%以上。	地方人民政府负责。	人力资源社会保障部

17	劳动人事争议调解仲裁	存在劳动人事关系的用人单位和劳动者	提供劳动人事争议调解和仲裁服务，推动劳动人事争议调解成功率达到60%以上，仲裁案件结案率达到90%以上。	地方人民政府负责。	人力资源社会保障部

18	劳动保障监察	各类用人单位和劳动者	提供法律咨询和执法维权服务。	地方人民政府负责。	人力资源社会保障部

三、基本社会保险

19	职工基本养老保险	符合条件的参保退休人员	发放基本养老金，包括基础养老金和个人账户养老金，对改革前参加工作、改革后退休的参保人员增发过渡性养老金，建立基本养老金合理调整机制。	用人单位原则上缴纳工资总额的20%，职工缴纳本人缴费工资的8%。在基本养老保险基金中支出，基本养老保险基金支付不足时财政给予补助。	人力资源社会保障部

20	城乡居民基本养老保险	符合条件的城乡居民	发放基础养老金和个人账户养老金。目前，国家确定的基础养老金最低标准为每人每月70元。根据经济发展和物价变动等情况，建立基础养老金水平合理调整机制。	在基本养老保险基金中支出。国家确定基础养老金最低标准。中央财政对中西部地区按国家确定的基础养老金标准给予全额补助，对东部地区给予50%补助。地方人民政府对参保人缴费给予补贴。	人力资源社会保障部、财政部

21	职工基本医疗保险	职工、无雇工的个体工商户、非全日制从业人员及灵活就业人员	政策范围内住院费用医保基金支付比例稳定在75%左右。	用人单位缴纳工资总额的6%左右，职工缴纳本人缴费工资的2%。具体缴费比例由各统筹地区规定。	人力资源社会保障部

22	生育保险	各类企业、机关、事业单位、社会团体等用人单位	基金支付生育期间的医疗费和生育津贴，生育津贴按职工所在用人单位上年度职工月平均工资计发。	用人单位按照不超过工资总额1%的比例缴纳生育保险费，累计结余超过9个月的统筹地区，应将费率控制在用人单位工资总额的0.5%以内。具体缴费比例由各统筹地区规定。	人力资源社会保障部

23	城乡居民基本医疗保险	除职工基本医疗保险应参保人员以外的其他所有城乡居民（包括农村人口和城镇非就业人员）	整合城镇居民基本医疗保险和新型农村合作医疗保险，政策范围内住院费用医保基金支付比例稳定在75%左右，大病保险的报销比例达到50%以上。	个人缴费和政府补助相结合。	人力资源社会保障部、国家卫生计生委、财政部

24	失业保险	依法参保并足额缴纳失业保险费的用人单位及其职工、失业人员	对符合条件的失业人员支付失业保险金、基本医疗保险费、丧葬补助金和抚恤金等，对符合条件的企业给予各类稳定岗位补贴。参保人数在1.8亿人左右。	按照《失业保险条例》，城镇企业事业单位按照本单位工资总额的2%缴纳失业保险费，职工按照本人缴费工资的1%缴纳失业保险费，农民合同制工人本人不缴纳失业保险费。按照《人力资源社会保障部 财政部关于阶段性降低社会保险费率的通知》（人社部发〔2016〕36号），从2016年5月1日起，失业保险总费率在2015年已降低1个百分点基础上可阶段性降至1%-1.5%。其中个人费率不超过0.5%，降低费率的期限暂按两年执行。具体方案由各省（区、市）确定。	人力资源社会保障部

25	工伤保险	企业、事业单位、社会团体、民办非企业单位、基金会、律师事务所、会计师事务所等组织的职工和个体工商户的雇工	保障因工作遭受事故伤害或者患职业病的职工获得医疗救治和经济补偿，促进工伤预防和职业康复。工伤保险基金和用人单位按规定支付工伤医疗和康复费用、伤残津贴和补助、生活护理费及工亡补助等。参保人数达到2.2亿人以上。	工伤预防的宣传、培训等费用，劳动能力鉴定费用和工伤保险待遇费用依法由工伤保险基金和用人单位支付。	人力资源社会保障部

四、基本医疗卫生

26	居民健康档案	城乡居民	为辖区常住人口建立统一、规范的居民电子健康档案，建档率逐步达到90%。	地方人民政府负责，中央财政适当补助。	国家卫生计生委

27	健康教育	城乡居民	提供健康教育、健康咨询等服务。	地方人民政府负责，中央财政适当补助。	国家卫生计生委

28	预防接种	0-6岁儿童和其他重点人群	在重点地区，对重点人群进行针对性接种国家免疫规划疫苗。以乡镇（街道）为单位，适龄儿童免疫规划疫苗接种率逐步达到90%以上。	地方人民政府负责，中央财政适当补助。	国家卫生计生委

29	传染病及突发公共卫生事件报告和处理	法定传染病病人、疑似病人、密切接触者和突发公共卫生事件伤病员及相关人群	就诊的传染病病例和疑似病例以及突发公共卫生事件伤病员及时得到发现、登记、报告、处理，提供传染病防治和突发公共卫生事件防范知识宣传和咨询服务。传染病报告率和报告及时率均达到95%，突发公共卫生事件相关信息报告率达到100%。	地方人民政府负责，中央财政适当补助。	国家卫生计生委

30	儿童健康管理	0-6岁儿童	提供新生儿访视、儿童保健系统管理、体格检查、儿童营养与喂养指导、生长发育监测及评价和健康指导等服务。0-6岁儿童健康管理率逐步达到90%。	地方人民政府负责，中央财政适当补助。	国家卫生计生委

31	孕产妇健康管理	孕产妇	提供孕期保健、产后访视及健康指导服务。孕产妇系统管理率逐步达到90%以上。	地方人民政府负责，中央财政适当补助。	国家卫生计生委

32	老年人健康管理	65岁及以上老年人	提供生活方式和健康状况评估、体格检查、辅助检查和健康指导等健康管理服务。65岁及以上老年人健康管理率逐步达到70%。	地方人民政府负责，中央财政适当补助。	国家卫生计生委

33	慢性病患者管理	原发性高血压患者和Ⅱ型糖尿病患者	提供登记管理、健康指导、定期随访和体格检查服务。全国计划管理高血压患者约1亿人，糖尿病患者约3500万人。	地方人民政府负责，中央财政适当补助。	国家卫生计生委

34	严重精神障碍患者管理	严重精神障碍患者	提供登记管理、随访指导服务。在册患者管理率和精神分裂症治疗率逐步均达到80%以上。	地方人民政府负责，中央财政适当补助。	国家卫生计生委

35	卫生计生监督协管	城乡居民	提供食品安全信息报告、饮用水卫生安全巡查、学校卫生服务、非法行医和非法采供血信息报告等服务。逐步覆盖90%以上的乡镇。	地方人民政府负责，中央财政适当补助。	国家卫生计生委

36	结核病患者健康管理	辖区内确诊的肺结核患者	提供肺结核筛查及推介转诊、入户随访、督导服药、结果评估等服务。结核病患者健康管理服务率逐步达到90%。	地方人民政府负责，中央财政适当补助。	国家卫生计生委

37	中医药健康管理	65岁以上老人、0-3岁儿童	通过基本公共卫生服务项目为65岁以上老人提供中医体质辨识和中医保健指导服务，为0-3岁儿童提供中医调养服务。目标人群覆盖率逐步达到65%。	地方人民政府负责，中央财政适当补助。	国家卫生计生委、国家中医药局

38	艾滋病病毒感染者和病人随访管理	艾滋病病毒感染者和病人	在医疗卫生机构指导下，为艾滋病病毒感染者和病人提供随访服务。感染者和病人规范管理率逐步达到90%。	地方人民政府负责，中央财政适当补助。	国家卫生计生委、国家中医药局

39	社区艾滋病高危行为人群干预	艾滋病性传播高危行为人群	为艾滋病性传播高危行为人群提供综合干预措施。干预措施覆盖率逐步达到90%。	地方人民政府负责，中央财政适当补助。	国家卫生计生委

40	免费孕前优生健康检查	农村计划怀孕夫妇	提供健康教育、健康检查、风险评估和咨询指导等孕前优生服务。目标人群覆盖率逐步达到80%。	中央和地方财政按比例分担。	国家卫生计生委

41	基本药物制度	城乡居民	政府办基层医疗卫生机构全部实行基本药物零差率销售，按规定纳入基本医疗保险药品报销目录，逐步提高实际报销水平。	地方人民政府负责，中央财政适当补助。	国家卫生计生委

42	计划生育技术指导咨询	育龄人群	提供计划生育技术指导咨询服务、计划生育相关的临床医疗服务、符合条件的再生育技术服务和计划生育宣传服务。	农村避孕节育技术服务经费由地方财政保障，中央财政对西部困难地区给予补助。	国家卫生计生委、财政部

43	农村部分计划生育家庭奖励扶助	年满60周岁、只生育一个子女或两个女孩的农村计划生育家庭夫妇	发放一定数额的奖励扶助金，并根据经济社会发展水平实行奖励扶助标准动态调整。	中央和地方财政按比例共同负担。	国家卫生计生委、财政部

44	计划生育家庭特别扶助	符合条件的独生子女伤残、死亡的父母及节育手术并发症三级以上人员	根据不同情况，给予适当扶助，并根据经济社会发展水平实行特别扶助标准动态调整。	中央和地方财政按比例共同负担。	国家卫生计生委、财政部

45	食品药品安全保障	城乡居民	对供应城乡居民的食品药品开展监督检查，及时发现并消除风险。对药品医疗器械实施风险分类管理，提高对高风险对象的监管强度。	中央和地方人民政府分类负责。	食品药品监管总局

五、基本社会服务

46	最低生活保障	家庭成员人均收入低于当地最低生活保障标准，且符合当地最低生活保障家庭财产状况规定的家庭	按照共同生活的家庭成员人均收入低于当地最低生活保障标准的差额，按月发给最低生活保障金。	地方人民政府负责，中央财政对困难地区适当补助。	民政部、财政部

47	特困人员救助供养	无劳动能力、无生活来源且无法定赡养、抚养、扶养义务人，或者其法定义务人无赡养、抚养、扶养能力的老年人、残疾人以及未满16周岁的未成年人	提供基本生活条件；对生活不能自理的给予照料；提供疾病治疗；办理丧葬事宜；对符合规定标准的住房困难的分散供养特困人员，给予住房救助；对在义务教育阶段就学的特困人员，给予教育救助；对在高中教育（含中职）、普通高等教育阶段就学的特困人员，根据实际情况给予适当教育救助。	地方人民政府负责，中央财政对困难地区适当补助。	民政部、财政部

48	医疗救助	重点救助对象：最低生活保障家庭成员和特困救助供养人员。

低收入救助对象：低收入家庭的老年人、未成年人、重度残疾人和重病患者，以及其他特殊困难人员。

重特大疾病医疗救助对象：除上述救助对象以外，还包括因病致贫家庭重病患者。

疾病应急救助对象：在中国境内发生急重危伤病、需要急救但身份不明确或无力支付相应费用的患者。	对重点救助对象参加城乡居民基本医疗保险的个人缴费部分进行补贴，对特困救助供养人员给予全额资助，对最低生活保障家庭成员给予定额资助。重点救助对象在定点医疗机构发生的政策范围内住院费用中，对经过基本医疗保险、城乡居民大病保险及各类补充医疗保险、商业保险报销的个人负担费用，在年度救助限额内按不低于70%的比例给予救助。对重点救助对象和低收入救助对象经基本医疗保险、城乡居民大病保险及各类补充医疗保险、商业保险等报销后个人负担的合规医疗费用，直接予以补助；因病致贫家庭重病患者等其他救助对象负担的合规医疗费用，先由其个人支付，对超过家庭负担能力的部分予以救助。医疗机构对疾病应急救助对象紧急救治所发生的费用，可向疾病应急救助基金申请补助。	地方人民政府负责，中央财政适当补助。	民政部、国家卫生计生委、财政部

49	临时救助	家庭对象：因火灾、交通事故等意外事件，家庭成员突发重大疾病等原因，导致基本生活暂时出现严重困难的家庭；因生活必需支出突然增加超出家庭承受能力，导致基本生活暂时出现严重困难的最低生活保障家庭；遭遇其他特殊困难的家庭。

个人对象：因遭遇火灾、交通事故、突发重大疾病或其他特殊困难，暂时无法得到家庭支持，导致基本生活陷入困境的个人。	为救助对象发放临时救助金；根据临时救助标准和救助对象基本生活需要，发放衣物、食品、饮用水，提供临时住所；对给予临时救助金、实物救助后，仍不能解决临时救助对象困难的，可分情况提供转介服务。县级以上地方人民政府根据救助对象困难类型、困难程度，统筹考虑其他社会救助制度保障水平，合理确定临时救助标准，并适时调整。	地方人民政府负责，中央财政对困难地区适当补助。	民政部、财政部

50	受灾人员救助	基本生活受到自然灾害严重影响的人员	及时为受灾人员提供必要的食品、饮用水、衣被、取暖、临时住所、医疗防疫等应急救助；对住房损毁严重的受灾人员进行过渡性安置；及时核实本行政区域内居民住房恢复重建补助对象，并给予资金、物资等救助；受灾地区人民政府应当为因当年冬寒或者次年春荒遇到生活困难的受灾人员提供基本生活救助。	中央和地方人民政府共同负责。	民政部、财政部

51	法律援助	经济困难公民和特殊案件当事人	提供必要的法律咨询、代理、刑事辩护等无偿法律服务。	地方人民政府负责，中央财政引导地方加大投入力度。	司法部、财政部

52	老年人福利补贴	经济困难的高龄、失能老年人	对经济困难的高龄老年人，逐步给予养老服务补贴；对生活长期不能自理、经济困难的老年人，给予护理补贴。	地方人民政府负责。	民政部、财政部

53	困境儿童保障	因家庭贫困导致生活、就医、就学等困难的儿童，因自身残疾导致康复、照料、护理和社会融入等困难的儿童，以及因家庭监护缺失或监护不当遭受虐待、遗弃、意外伤害、不法侵害等导致人身安全受到威胁或侵害的儿童	为困境儿童提供基本生活、基本医疗、教育等服务，落实监护责任。各地统筹考虑困境儿童的困难类型、困难程度、致困原因，完善落实社会救助、社会福利等保障政策。	地方人民政府负责。	民政部、财政部

54	农村留守儿童关爱保护	父母双方外出务工或一方外出务工另一方无监护能力、未满16周岁的农村户籍未成年人	强化家庭监护主体责任；落实县、乡镇人民政府和村（居）民委员会职责；加大教育部门和学校关爱保护力度；动员群团组织开展关爱服务；推动社会力量积极参与。	地方人民政府负责。	民政部

55	基本殡葬服务	执行国家殡葬政策的困难群众	为城乡困难群众以减免费用或补贴方式提供遗体接运、暂存、火化、骨灰寄存等基本殡葬服务；为优抚对象及城乡困难群众免费或低收费提供骨灰节地生态安葬服务。	地方人民政府负责。	民政部、财政部

56	优待抚恤	享受国家抚恤补助的优抚人员	建立完善优抚对象待遇与贡献相一致的优抚保障体系，将优抚对象优先纳入覆盖一般群众的救助、养老、医疗、住房以及残疾人保障等各项社会保障制度体系。	中央和地方人民政府分级负担。	民政部、财政部

57	退役军人安置	退役军人	自主就业的，在领取退役金后，按规定享受扶持就业优惠政策；其他分别采取安排工作、退休、供养等方式予以安置。	中央和地方人民政府共同负责。	民政部、财政部

58	重点优抚对象集中供养	需要常年医疗或者独身一人不便分散安置的一级至四级残疾退役军人；老年、残疾或者未满16周岁的烈士遗属、因公牺牲军人遗属、病故军人遗属和进入老年的残疾军人、复员军人、退伍军人中无法定赡养人（扶养人、抚养人）或赡养人（扶养人、抚养人）无赡养（扶养、抚养）能力且享受国家定期抚恤补助待遇的优抚对象	建立完善优抚对象待遇与贡献相一致的优抚保障体系，依托优抚医院、光荣院，给予符合条件的重点优抚对象集中供养、医疗等保障。	中央和地方人民政府共同负责。	民政部、财政部

六、基本住房保障

59	公共租赁住房	符合条件的城镇低收入住房困难家庭、城镇中等偏下收入住房困难家庭、新就业无房职工、城镇稳定就业的外来务工人员	实行实物保障与货币补贴并举，并逐步加大租赁补贴发放力度。	市、县级人民政府负责，引导社会资金投入，省级人民政府给予资金支持，中央财政给予资金补助。	住房城乡建设部、财政部

60	城镇棚户区住房改造	符合条件的城镇居民	实物安置和货币补偿相结合，具体标准由市、县级人民政府确定（有国家标准的，执行国家标准）。全国开工改造包括城市危房、城中村在内的各类棚户区住房2000万套。	政府给予适当补助，企业安排一定的资金，住户承担一部分住房改善费用。	住房城乡建设部、财政部

61	农村危房改造	居住在危房中的建档立卡贫困户、分散供养特困人员、低保户、贫困残疾人家庭等贫困农户	支持符合条件的贫困农户改造危房，各省份确定不同地区、不同类型、不同档次的省级分类补助标准，中央财政给予适当补助，基本完成存量危房改造任务。地震设防地区结合危房改造，统筹开展农房抗震改造。	地方人民政府负责，中央财政安排补助资金、地方财政给予资金支持、个人自筹等相结合。	住房城乡建设部、财政部

七、基本公共文化体育

62	公共文化设施免费开放	城乡居民	公共图书馆、文化馆（站）、公共博物馆（非文物建筑及遗址类）、公共美术馆等公共文化设施免费开放，基本服务项目健全。	地方人民政府负责，中央财政适当补助。	文化部、国家文物局、财政部

63	送地方戏	农村居民	根据群众实际需求，采取政府购买服务等方式，为农村乡镇每年提供戏曲等文艺演出服务。	地方人民政府负责，中央财政适当补助。	文化部、教育部、新闻出版广电总局、财政部

64	收听广播	城乡居民	为全民提供突发事件应急广播服务。通过直播卫星提供不少于17套广播节目，通过无线模拟提供不少于6套广播节目，通过数字音频提供不少于15套广播节目。	中央和地方人民政府共同负责。	新闻出版广电总局、财政部

65	观看电视	城乡居民	通过直播卫星提供25套电视节目，通过地面数字电视提供不少于15套电视节目，未完成无线数字化转换的地区提供不少于5套电视节目。	中央和地方人民政府共同负责。	新闻出版广电总局、财政部

66	观赏电影	农村居民、中小学生	为农村群众提供数字电影放映服务，其中每年国产新片（院线上映不超过2年）比例不少于1/3。为中小学生每学期提供2部爱国主义教育影片。	地方人民政府负责，中央财政适当补助。	新闻出版广电总局、财政部

67	读书看报	城乡居民	公共图书馆（室）、文化馆（站）和行政村（社区）综合文化服务中心（含农家书屋）等配备图书、报刊和电子书刊，并免费提供借阅服务；在城镇主要街道、公共场所、居民小区等人流密集地点设置公共阅报栏（屏），提供时政、“三农”、科普、文化、生活等方面的信息服务。	地方人民政府负责，中央财政适当补助。	文化部、新闻出版广电总局、财政部

68	少数民族文化服务	主要少数民族地区居民	通过有线、无线、卫星等方式提供民族语言广播影视节目；提供民族语言文字出版的、价格适宜的常用书报刊、电子音像制品和数字出版产品。提供少数民族特色的艺术作品，开展少数民族文化活动。	地方人民政府负责，中央财政对部分事项予以补助。	新闻出版广电总局、文化部、财政部

69	参观文化遗产	未成年人、老年人、现役军人、残疾人和低收入人群	参观文物建筑及遗址类博物馆实行门票减免，文化和自然遗产日免费参观。	中央和地方财政分别负担。	国家文物局、财政部

70	公共体育场馆开放	城乡居民	有条件的公共体育设施免费或低收费开放；推进学校体育设施逐步向公众开放。	地方人民政府负责，中央财政对部分事项予以补助。	体育总局、教育部、财政部

71	全民健身服务	城乡居民	提供科学健身指导、群众健身活动和比赛、科学健身知识等服务；免费提供公园、绿地等公共场所全民健身器材。	地方人民政府负责，中央财政对部分事项予以补助。	体育总局、教育部、财政部

八、残疾人基本公共服务

72	困难残疾人生活补贴和重度残疾人护理补贴	困难残疾人和重度残疾人	为低保家庭中的残疾人提供生活补贴，为残疾等级被评定为一级、二级且需要长期照护的重度残疾人提供护理补贴。有条件的地方可逐步提高补贴标准、扩大补贴范围。	地方人民政府负责，中央财政适当补助。	民政部、财政部、中国残联

73	无业重度残疾人最低生活保障	生活困难、靠家庭供养且无法单独立户的成年无业重度残疾人	经个人申请，可按照单人户纳入最低生活保障范围。	地方人民政府负责，中央财政适当补助。	民政部、中国残联

74	残疾人基本社会保险个人缴费资助和保险待遇	贫困和重度残疾人	为参加居民基本养老保险、居民基本医疗保险的服务对象按规定提供个人缴费补贴；将符合规定的医疗康复项目、基本的治疗性康复辅助器具逐步纳入基本医疗保障范围。	缴费资助由地方人民政府负责或医疗救助基金支出；报销由基本医疗保险基金支出。	人力资源社会保障部、民政部、国家卫生计生委、中国残联

75	残疾人基本住房保障	残疾人	对符合基本住房保障条件的城镇残疾人家庭给予优先轮候、优先选房等政策；同等条件下优先为经济困难的残疾人家庭实施农村危房改造，完成农村贫困残疾人家庭存量危房改造任务。	由地方人民政府负责，中央财政安排补助资金、地方财政给予资金支持、个人自筹等相结合。	住房城乡建设部、中国残联

76	残疾人托养服务	就业年龄段智力、精神及重度肢体残疾人	支持日间照料机构和专业托养服务机构为100万残疾人提供护理照料、生活自理能力和社会适应能力训练、职业康复、劳动技能培训、辅助性就业等服务。	地方人民政府负责，中央财政适当补助。	中国残联、财政部

77	残疾人康复	有康复需求的持证残疾人、残疾儿童	提供康复建档、评估、训练、心理疏导、护理、生活照料、辅具适配、咨询、指导和转介等基本康复服务；开展残疾儿童康复救助，逐步为0-6岁视力、听力、言语、智力、肢体残疾儿童和孤独症儿童免费提供手术、辅助器具配置和康复训练等服务。	地方人民政府负责，中央财政适当补助。	中国残联、国家卫生计生委、民政部

78	残疾人教育	残疾儿童、青少年	逐步为家庭经济困难的残疾学生提供包括义务教育、高中阶段教育在内的12年免费教育；对残疾儿童普惠性学前教育予以资助；对残疾学生特殊学习用品、教育训练、交通费等予以补助。	地方人民政府负责，中央财政适当补助。	财政部、教育部、中国残联

79	残疾人职业培训和就业服务	有劳动能力和就业意愿的城乡残疾人	各级公共就业服务机构及残疾人就业服务机构按规定为城镇残疾人提供有针对性的职业技能培训、岗位技能提升培训、创业培训等就业创业服务；为50万中西部地区农村贫困残疾人提供农业实用技术培训。	地方人民政府负责，中央财政适当补助。	中国残联、人力资源社会保障部、农业部

80	残疾人文化体育	残疾人	能够收看到有字幕或手语的电视节目，在公共图书馆得到盲文和有声读物等阅读服务；为基层残疾人体育活动场所和残疾人综合服务设施配置适宜的器材器械。	地方人民政府负责，中央财政适当补助。	中国残联、文化部、新闻出版广电总局、体育总局

81	无障碍环境支持	残疾人、老年人等	推进公共场所和设施无障碍改造；对贫困重度残疾人家庭继续开展无障碍改造；逐步开展互联网和移动互联网无障碍信息服务。	地方人民政府负责。	住房城乡建设部、工业和信息化部、中国残联

　　附件2

　　重点任务分工方案

序号	重　点　任　务	责　任　单　位

1	完善国家基本公共服务制度，建立基本公共服务清单制，建立健全科学有效的基本公共服务实施机制。	国家发展改革委牵头，其他有关部门按职责分工负责

2	推动基本公共教育领域发展指标、重点任务、保障措施有效落实。	教育部牵头，其他有关部门按职责分工负责

3	推动基本劳动就业创业领域发展指标、重点任务、保障措施有效落实。	人力资源社会保障部牵头，其他有关部门按职责分工负责

4	推动基本社会保险领域发展指标、重点任务、保障措施有效落实。	人力资源社会保障部牵头，其他有关部门按职责分工负责

5	推动基本医疗卫生领域发展指标、重点任务、保障措施有效落实。	国家卫生计生委、食品药品监管总局、国家中医药局分别牵头，其他有关部门按职责分工负责

6	推动基本社会服务领域发展指标、重点任务、保障措施有效落实。	民政部牵头，其他有关部门按职责分工负责

7	推动基本住房保障领域发展指标、重点任务、保障措施有效落实。	住房城乡建设部牵头，其他有关部门按职责分工负责

8	推动基本公共文化体育领域发展指标、重点任务、保障措施有效落实。	文化部、新闻出版广电总局、体育总局、国家文物局分别牵头，其他有关部门按职责分工负责

9	推动残疾人基本公共服务领域发展指标、重点任务、保障措施有效落实。	中国残联牵头，其他有关部门按职责分工负责

10	开展贫困地区脱贫攻坚。	国务院扶贫办牵头，其他有关部门按职责分工负责

11	重点帮扶特殊困难人群。	民政部牵头，其他有关部门按职责分工负责

12	促进基本公共服务城镇常住人口全覆盖。	公安部牵头，其他有关部门按职责分工负责

13	缩小城乡基本公共服务差距，提高区域服务均等化水平，夯实基层服务基础。	国家发展改革委、财政部牵头，其他有关部门按职责分工负责

14	加快事业单位分类改革，理顺政府与事业单位在基本公共服务供给中的关系。	中央编办牵头，其他有关部门按职责分工负责

15	大力发展社会组织，支持其承接基层基本公共服务和政府委托事项。	民政部牵头，其他有关部门按职责分工负责

16	推进政府购买公共服务。	财政部牵头，其他有关部门按职责分工负责

17	积极引导社会力量参与基本公共服务供给，加强政府和社会资本合作。	财政部、国家发展改革委牵头，其他有关部门按职责分工负责

18	鼓励发展志愿和慈善服务，扩大基本公共服务供给。	民政部牵头，其他有关部门按职责分工负责

19	加大财政对基本公共服务的投入力度，优化转移支付结构，提高资金使用效率。	财政部牵头，其他有关部门按职责分工负责

20	加强公共服务人才培养培训。	教育部、人力资源社会保障部牵头，其他有关部门按职责分工负责

21	促进公共服务人才合理流动，提升基层人员能力。	人力资源社会保障部牵头，其他有关部门按职责分工负责

22	加强公共服务设施规划布局和用地保障。	住房城乡建设部、国土资源部牵头，其他有关部门按职责分工负责

23	建立健全公共服务标准体系。	质检总局牵头，其他有关部门按职责分工负责

24	加强公共服务行业自律和社会监督，强化社会信用体系支撑。	国家发展改革委牵头，其他有关部门按职责分工负责

25	建立健全基本公共服务综合评估指标体系，推进统计信息库建设，开展年度统计监测。	国家发展改革委、国家统计局牵头，其他有关部门按职责分工负责

26	组织规划评估，加强绩效评价和监督问责。	国家发展改革委牵头，地方各级人民政府和其他有关部门按职责分工负责
